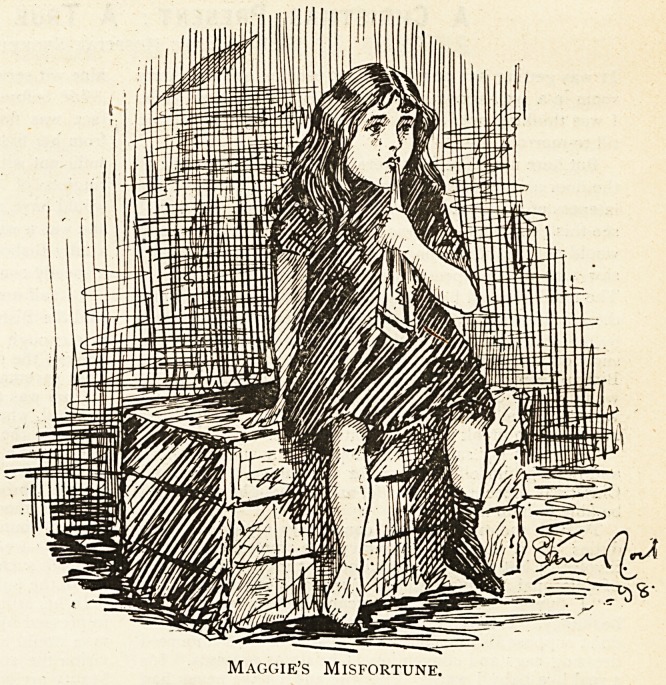# The Hospital.—Christmas Appeal Supplement

**Published:** 1898-12-17

**Authors:** 


### CHRISTMAS APPEAL SUPPLEMENT—ILLUSTRATED.

### Christmas and the Hospitals.

As one Christmas comes round after another each year offers new problems for solution. Yet among the ever-varying topics which occupy men's minds there is one which is always present. Just as the pattern in the kaleidoscope may change at every move, and yet the same bits of glass may be found in every image, so, underlying all the various social problems which crop up for discussion, there is always the great problem of poverty, and the great question what to do with those who, poor even when at work, are brought to the verge of destitution when sickness deprives them of their income. All this would be true of any period of the year, but its consideration is especially appropriate at Christmas, when men's minds are bent on charity, when purse-strings are unloosed, and when for once men put in practice what they hear so often preached, and feel drawn to do to others as they would be done by.

Still, the matter is not exclusively one of charity and good feeling. At the bottom of the problem are great social questions. To a very large extent, to a much larger extent in fact than many people imagine, the stability of our present social system depends upon those great annual outpourings of charity, those free gifts offered by the fortunate to the unfortunate which are peculiarly associated with Christmas time, and with all that it celebrates and connotes. By aid of these gifts hospitals are maintained, and thus life is made bearable to an immense number of people who, without such assistance, would find the present condition of affairs absolutely intolerable. Those who plunge into the inner life of London, and investigate the homes, such as they are, in which a large proportion of the inhabitants of London live, are apt to come away appalled, and to ask themselves, as indeed they well may, What is there to keep together a social fabric in which so large a number obtain so little in return for their life's work? The question is an anxious one, and suggests many very unpleasant and dangerous possibilities. In view of the facts of London life one must admit the immense importance of every message of peace and good-will from class to class, and in view of the work that is being done by the London hospitals among the London poor, one must admit that in addition to what they do in saving life, in curing disease, and in relieving suffering, they also do a great and important, though little recognised, work in promoting that contentment among the workers on which hinges much of our prosperity. Just as we write, the problem which is most prominently before the public is that of the housing of the working classes, and without here saying a word as to the action which has been proposed for alleviating the present pressure, this at least must be taken as proved, that the difficulty of obtaining house room in central London is a great and urgent one, and that but for the relief afforded by the hospitals the present condition of affairs would be full of danger both to the healthy and the sick. The causes of the present condition of overcrowding and its remedies are matters for the economists and for our elected rulers. But what is obvious to the philanthropist, and, indeed, to every ordinary person who wishes well to his neighbour, is that so difficult is it to obtain a home, and so great is the demand for rooms, that families who must live near their work are driven to crowd together into such small space that there is absolutely no room left for those who may happen to be ill. It is, to a large extent, this lack of house room, and the fact that they, the workers' homes in the central parts of London, are crammed as full of people as they will hold, that makes the maintenance of the hospitals so absolutely essential. It is not altogether a matter of paying the doctor, nor of obtaining the necessaries of life, nor even of nursing the invalid. All these are great difficulties, no doubt, but the poor are wonderfully ready to help each other in sickness; and, although it would be impossible to bring into the homes of the poor the highest scientific treatment, something at least might be done to alleviate suffering, if only there was room. But there is no room. Visit these houses in the daytime and things may not appear so bad, but at night, when all are in and are packed away in bed, then one sees that these homes are no places for the sick, and that—if anything like cure is to be aimed at—these unfortunates must be removed at once to the hospital. This, we take it, is, and always will be, the crux of the whole question. The hospitals are useful and helpful in many ways and for many reasons, but they are essential because there is not room at home. But this is not everything.

### Science and the Slums.

We live in an age of progress, and the progress of science has made the medicine and surgery of to-day absolutely unlike anything that the world has seen before. Time was when charity was a much simpler affair than it is to-day. The Lady Bountiful could go down into her district and from her kitchen she could supply soup, and food, and wine, and she could send her own doctor, who, in turn, could supply just the same sort of physic in just the same sort of bottles as he would send to Lady Bountiful herself.

Slumming, then, was a most effectual performance, for by dint of a few presents the slums were made equal to the palace. When only two wants were recognised, that of food and that of physic, it was not difficult for the rich man, whose kitchen and whose doctor were at the service of the poor, to feel that he had done his duty to his neighbour, for by their aid he could place his sick dependent in the best known position for recovery.

Increased knowledge has, however, made all this impossible. No amount of "delicacies," or of "doctor's stuff" will put a patient, whose home is in a crowded slum, into the best known position for recovery. The certainty has been borne in upon us that much of the disease from which the poor suffer is the outcome of the surroundings in which they live, and that the attempt to cure them while these surroundings exist is like baling water into a sieve. No. The Lady Bountiful may visit in the slums, and by her sympathy and her purse may help the healthy, but so far as the sick are concerned she does her best, not by gifts, but by carrying the invalid away from his evil surroundings and placing in a hospital in which everything is arranged for the treatment of disease. Let there be no mistake about this. When we have to do with the poor, and especially with the overcrowded poor, as they exist in London, no man by mere impulsive giving can obtain the gratification of feeling that even one sick person has been placed in the best known position for recovery. This can only be done by supporting the hospitals. And would it not be a pleasant and a comfortable thing, a thing that should make a man enjoy his Christmas dinner with greater zest, and look round on his own home with greater satisfaction, to feel that by his gift to a neighbouring hospital he has lifted some sick person out of the depths, and placed some invalid in "the best known position for recovery."

This is a plan of giving which we strongly recommend: Support a bed in some hospital for a year; visit that bed every week and see what work it does and what a variety of misery it relieves before another Christmas comes round; and then at next year's Christmas dinner tell the story—it will be by far the most interesting story of the evening.

### Commerce and the Hospitals.

In the social system of every community there clearly must be some receptacle for the social residuum, for those who are too old for work, for those who are too weak, whether in mind or body, to earn their living, and for such of the sick as have nowhere else to go to, and such a receptacle is the workhouse. In a state of savagery these weaklings would quickly die, but, in accordance with the dictates of Christianity and humanity, we keep them alive, paying rates annually for the purposes, and there are a few rigid disciples of political economy who, even at Christmas time, will say, "Why should I give to hospitals? I have borne my share in supporting the workhouse, and when I have paid my poor's-rate, I think I have done my duty towards my neighbour," and indeed if things were all perfectly and harmoniously arranged in this sublunary sphere, if those who are rich were always rich, and those who are poor were always poor, so poor as to be well cared for by the guardians, however unjust such an arrangement might appear, at least it would work with considerable simplicity. The rich would pay their rates and the poor would eat their bread and butter, and there would be nothing more to be said.

Things, however, are not quite so simple. According to the oscillations of the market, and the constantly varying demand for labour, workers who are sometimes well off are at other times on the verge of destitution. "Bad times" mean to some people a grouse moor the less, or a shorter visit to the seaside or some such thing. To others, however, it means a complete cessation of wages for many weeks, the gradual dispersion of the little home, and absolute destitution. This is one of the terrible hardships which of necessity accompanies a world-wide commerce, and it is this constant fluctuation in the labour market which makes it impossible to draw a rigid line between the poor and the well-to-do. But if we cannot help all this; if, as seems to be the case, it is an inexorable law that the final stress and strain of a fluctuating commerce comes upon the workers, we can at least do something to help their sick. This is one of the great claims which the hospitals have upon a wealthy city which lives by commerce, namely, that every year large numbers of deserving people are thrown out of employment, and brought from comfort to poverty, by no fault of their own, but by the direct operation of the trade and commerce by which the wealth of London is produced. To the healthy this is bad enough, involving much hardship and much loss, but to the sick it means death or the workhouse, unless some of the wealth which commerce makes is handed back to the support of great hospitals in which these helpless victims of trade's iron hand may find succour, and, perchance, regain what is always the worker's most useful form of capital, namely, health and strength.

### The Moral.

We commend the hospitals of London, then, to the charity, the goodwill, and the personal care of all who dwell in London, or in London's suburbs, or who, wherever they may dwell, draw even a portion of their wealth from London work. But especially we appeal for charitable gifts and for personal interest. Visit the hospitals, find out the patients from your own neighbourhood, and see if you can help them on their return to their own life; become a subscriber to a hospital and get on the board, and take your share in the management of one at least of these great and absolutely necessary charities. But if you cannot do these things, at least there is one thing that all can do at Christmas time—among your many Christmas gifts send something to the hospitals.

### The Poorest of London Hospitals.

The Metropolitan Hospital, situated in the Kingsland Road, N.E., is probably more urgently in need of money at the present time than any other metropolitan hospital. Indeed, its financial condition is such that, unless the public promptly come forward with adequate resources, it will soon be necessary to close it altogether. With accommodation for 160 beds, no less than 72 are vacant at the present time for want of funds. It labours under two great disadvantages: (1) It is situated in a densely crowded district, out of sight of the wealthy; and (2) this absence of money has made it impossible for the management to give the interior that smart and bright appearance which ought to prevail in a modern hospital. The hospital is at present £7,000 in debt to its tradesmen, and is obliged to obtain everything on credit. Might we ask one or two of our wealthier readers to take the train to Dalston during Christmas week and visit the Metropolitan Hospital. The Secretary, Mr. Charles E. Byers, would give them a hearty welcome, and through this visit they could not fail to realise and appreciate the real needs of the poorest of London hospitals, which is situated in one of the most crowded districts of North-East London. It is essential in our view that the management should invite the co-operation of a few of those most interested in the metropolitan hospitals, and summon them to a conference, at which the whole system of administration and the present needs of the hospital can be thoroughly gone into. Such a step, if taken, should speedily produce all the money which is so urgently required. Few thoughtful people, who feel the responsibility of wealth as well as its advantages, can fail to be moved to give to this hospital in the distressing circumstances here stated.

### The Christmas Dinner to the Convalescents.

"The convalescents!" What a world of meaning is wrapped up in the title. Which Sister doesn't take an honest pride in her convalescents? And what wouldn't she do to further their interests and legitimate enjoyment? One may well say "legitimate" enjoyment, for they are such a careless, heedless, happy-go-lucky lot! They imagine that, having "pulled through all right," they are at liberty to return at once to the old habits and customs of rude health. No sooner was the Christmas Day dinner mooted in the male wards than sisters and nurses were almost driven crazy with the scores of wild suggestions and ventured hopes. One had never known what it was to have a Christmas dinner without brandy sauce; another didn't think anything of the occasion unless "a whacking big turkey" adorned the board, and another would insist on the superiority of roast pork to all other dishes. The steward put matters as well as he could by explaining that it was hardly possible to please everyone, and by adding that they must wait and be patient.

At last the great day arrives. The fidgeting in morning chapel is disgraceful (so an old gentleman says). At twelve precisely the male convalescents file in. There is "No. 6" (he is known by the number of his bed) pointing with enthusiasm at the steaming dish being carried in by Nurse; and little "No. 2" is cheekily questioning and disputing with his left hand neighbour as to his capacity for pudding. What a sight that table was to them, after weary weeks in bed! The fruits and flowers and crackers and jellies, and "everyfink slap up bang to the knocker, I tell 'e," as "No. 14" said to his wife when she came in with the visitors at two o'clock.

### Some of the Patients.

"Wal, Nus, 'ow's our little Billy this mornin'?" asks Billy père. He has called at the hospital to inquire as to the conduct and progress of his son and heir, who has had a fall from the donkey cart and put out his shoulder. The particular nurse who has been questioned knows nothing of the case, and is not able to give the desired information, but she puts the good-natured man in the way of getting it and speeds away. All sorts and conditions of human beings are included in the multitude that daily call either as patients or inquirers at the hospital. There is Mrs. Muggs, whose husband is in one of the wards with a badly cut head. She wants to know "'ow 'e's gittin' on." She becomes confidential as Nurse says the good man's condition is critical, and it is learned for the first time that the cut head was sustained not in a fall downstairs, as was first of all suggested, but by sharp and sudden contact with a broken ale pot. There is Bobby Smithers, who "'as called to know wot day our Sarah will come aht." There's an old and straightforward 'bus driver, with his ancient high hat squashed down upon his ears, who has made a special journey to learn all he can about the poor lady "what fell down" his omnibus steps the previous day. There's Dick, the coster, who wants a visiting card for Sunday afternoon, when he intends giving up the opportunity of half a day's good business in order to sit beside his old pal Tommy Bell. Many and extraordinary are the sayings and drolleries in which the hospital callers indulge. Were space available, our readers' powers of belief would be taxed by some of the queer stories we could recount.

SOME OF THE PATIENTS.

### What the Public Sends.

The great big patient public sends funds and necessaries of all descriptions, and hospital authorities could not be more grateful than they are, but it also sometimes sends oddments and scraps that are fearful and wonderful. Let me particularise. A large hospital in London has recently been in sore need of all manner of articles of clothing for those poor and needy patients whose health has been restored, and who are ready for the outside world and work again, but who are minus a pair of stockings—for though a poorly man may enter a hospital without stockings to his feet he cannot in common decency be permitted to leave it in that pitiable condition, or without a hat or a pair of boots, or (when rheumatism has been the ailment) a pair of warm gloves. Now what can the matron do with a billycock hat that has the crown hanging by a thread, or with a pair of socks that require two hours of careful darning, or with a pair of gloves that have a couple of the fingers and a thumb half worn (or chewed) away? Would not the gift have been of twenty times more value had the donor taken the necessary repairs in hand? Does the donor think that the bustling nurses who have twelve hours a day duty have more time in which to repair clothing than she has? In all due respect, the answer must be an emphatic No! And such gifts as tiny Japan dolls, and painted and varnished animals, would not be sent in the place of more serviceable gifts did the kindly givers only dwell for a moment on the danger of putting the toys named into the little hands of an infant. What mother does not know that the small mites must be provided with something that it won't hurt to suck and that there is no chance of swallowing.

### What is Wanted.

The Matron has a nice big cupboard, and it is known to all and sundry about the hospital that the shelves of this cupboard ought to be stacked with warm woollens and flannels, but it not infrequently happens that the demand for the useful articles referred to seriously exceeds the supply. There are so many poor who are often ill chiefly for lack of proper food and clothing. So cold and starved are they that some internal organ of their system "goes wrong." They are admitted to the ward and placed in a cosy bed, and medical and nursing skill and attention are lavished upon them. Gradually health returns, and the day arrives when the doctor says that number six or number ten may take his, or her, discharge and return to work. So far so good. However, sister cannot let number six leave his bed and put on a pair of thin, un lined trousers when it is most necessary that the poor legs should be kept warm, nor, in the case of a woman, can a threadbare skirt be donned unless it is over thick warm petticoats. Therefore, off dashes sister to matron for the necessary raiment. When such articles are sent by thoughtful friends to matron she has them in hand to distribute, but when she has not them sent in she has not them to distribute. Many a good conscientious matron and sister is oftentimes ready to weep with compassion for the ill-clad ex-patients who must of necessity leave the wards for other urgent cases to come in. Will not all those among our readers who can possibly help in this most deserving cause do what they can to make fewer the sad and harrowing experiences connected with the matron's Samaritan cupboard?

### Help the Incurables.

The constitution of the Hospital Sunday Fund has rendered it impossible to make a grant from that fund to the British Home for Incurables, Crown Lane, Streatham, S.W. This is an additional reason for urging its claims at Christmas each year. One of the most encouraging days that we ever spent was occupied in the inspection of this excellent institution. It is beautifully situated, excellently constructed, and the comfort of the inmates is the first consideration of all connected with the institution. It is economically administered; it is fortunate in its matron, who modestly omits her name from the report, but who is probably the most suitable person for such a post that could be found. In visiting some homes for incurables we have been struck at times by an absence of sympathy towards the inmates, but at the British Home for Incurables sympathy and comfort are the watchwords of all who are connected with the charity. It is essentially a cheerful institution. Every patient has the air of being thoroughly at home and perfectly satisfied, a circumstance the more remarkable having regard to the hopelessness of the cases under treatment. There is a good tone everywhere, and all the inmates seem perfectly happy. As illustrating the care and thought which have been brought to bear upon promoting the happiness of the patients, we may mention that there is a rota of daily visitors who are regarded by the incurables as real friends. To the bedridden and the helpless the daily visit of a kindly friend from outside causes each day to pass pleasantly, and brings resignation and even joy to the most afflicted of the human race. We have said enough to show that all who have money to give at Christmas should certainly devote a portion of their offerings to the British Home for Incurables. It is a national charity, wholly unsectarian; it is doing a great work with splendid efficiency. The British Home is fortunate in having an able and energetic secretary in Mr. R. G. Salmond, devoted to its interests, whose untiring labour has won for him the lasting gratitude of the helpless inmates. We hope that Mr. Salmond may receive many cheques at 72, Cheapside, E.C., the City office of the institution, in the course of the next few weeks.

"These helpless ones our care and pity claim;
A grant from treasures of our heart and wealth
Will buy a world for them whose feeble aim
Oft point at little joys unknown to health."

### "Anxious Moments."

Annie Bagshaw is in the midst of a spell of very anxious moments. Her brother Jim, who supports her, along with the mother and two little sisters, by the profits he makes in trudging about all the week through "dahn in the Boro' wif a barrer," has sustained an accident in the crowded street. For a long time it has been Annie's custom to go daily at noon to an agreed spot with Jim's dinner carefully packed up in a basin covered with a plate, the whole tied up in a red handkerchief of a serviceable size. And while Jim would sit upon one of the shafts of his "barrer" and contentedly eat his midday meal, Annie would stand by and chat as to the progress of the morning's takings. Bent on this errand the other day, she turned a corner just in time to see a runaway horse dash into Jim's "shop"—as he himself describes it—knocking it over and its owner with it. Her terrified screams were only heightened as she saw a constable and several bystanders lift his helpless body and carry it to the pavement. His eyes were closed, and his moans suggested to her all sorts of terrible things. Would he know her if she spoke to him? Was he dying?

Very quickly he is carefully placed in an ambulance and removed to the casualty room of the neighbouring hospital. There he is put upon a couch and surrounded by a screen, behind which a couple of house-surgeons are examining him. And it is for their verdict that Annie so anxiously waits. Let us hope that nothing more serious than "bad bruises and severe shock" will be the message that she is commissioned to carry to the mother and little sisters at home.

### THE NURSE OF YESTERDAY.

DICKENS has described her. She was, as a rule, a good old soul, believing in comfort for herself and strange and gruesome remedies for her patients. If a dozen of the nurses of yesterday could return to us, and could persuade a modern hospital matron to allow them access to the wards, the chief noticeable feature of their work and their methods would be its unutterable slowness, to say nothing of their ignorance and thoughtless cruelty. What up-to-date surgeon could keep his temper with a nurse of yesterday? He would only throw his instrument at her, and stamp with rage upon the floor. The mystery and hampered movements of times long ago are abolished for ever—luckily for the patient. Advancement in one branch has made advancement necessary in all. It would not do to say that there were no good nurses in the old days. Far be it from us to suggest it. Nursing instinct has existed for centuries, but never has it reached the pitch that it has in the present day. Where is the institution to-day where a nurse may be seen with that absolutely contented expression, that rude independence of attitude, that "Don't worry me—I'm thinking" aspect. There is no smug satisfaction nowadays. When a success is recorded it is attributed to science, and a fresh conquest is thereupon undertaken. Nursing was done with heavy hands once; to-day it is done by magically light touch. The curls that hung about the old nurse's ears would to-day be done away with if for no other reason than that they might be the means of preventing the whispered word of a dying child being caught. To honesty and plodding we have added point and knowledge. Let us be thankful.

### THE NURSE OF TO-DAY.

SHE is alert, and takes herself seriously in general. She is ambitious, too, and has her eye on a matronship. To use an expressive Americanism, "there are no flies on her." It may, of course, be just possible that she is an exceptional case; that she became a nurse because she imagined that the costume would suit her face and figure; that she might fall in with a young and rising doctor who would lay his hand and heart at her feet; that she would command the sympathy—perhaps the envy—of a host of girl friends by reason of the stress and toil voluntarily undergone. But our nurse in the accompanying sketch is obviously too eager, too earnest, too full of real nursing feeling to be false. Her heart is in her work, and by saying that her heart is in her work we would wish to imply that so anxious for success and commendation is she that every patient who comes under her care benefits to an extent that would be impossible were she different in her views and aims. Will an unlooked-for kind word or tender attention advance her charge's spirits in the right direction? then it is unhesitatingly given. In her person she is scrupulously neat and correct. Her cap is seldom too far forward or too far back. Yet she is not over-nice in her discharge of the duties imposed by her calling; rough work comes her way often enough, and she surmounts it with an ease only attainable when there is real solid will power. Her voice, her bearing, her very eyes and hands, speak of hopefulness and progress.

### CHRISTMAS IN THE COUNTRY HOSPITAL.

By Sister Grace.

Eight happy Christmases I spent in a large provincial hospital, and so claim to know something of the work encountered there. I shall never forget how nervous I felt about my decorations the first year, nor how much money I wasted. It is true I had spent two such seasons in London, but a humble probationer, having no responsibility, feels a joyous disregard of all difficulties, and takes no thought for the morrow in the matter of materials and resources. I gazed upon my two large wards and thirty beds with a feeling akin to despair, and if I had not possessed a few disjointed, but nevertheless valuable, ideas gained in barracks, my mind would have been a blank. Tommy Atkins' main idea in such matters is "wreathing," writ large, and adorned with paper roses. Now this is an excellent base from which to start, and I thank Tommy for his instruction in the art of manufacture, as I have had to thank him often in my life for other kindnesses, courtesy, politeness, and good manners, all which I have encountered in a life spent much among soldiers. Deeply as I admire Tommy, I do not much care for his paper roses, and usually supplied their place with coloured lanterns, which have a most delightful effect hung upon thick ropes of evergreens, but great care must be taken to avoid all danger of fire.

Being always an emulator of the early bird, who is invariably rewarded by the discovery of a succulent worm, I generally began my Christmas preparations three months in advance. This will perhaps appear an excessive precaution, but I found it answered well, and I never felt myself unpleasantly pressed at the last.

Bearing in mind the nature of my wards, and that accidents and operations have not the good taste to avoid festive seasons, I liked to be well forward, and so, though my wards were usually the busiest in the building, I was generally enjoying a sensation of leisurely security, and appropriating complacently to myself the parable of the wise and foolish virgins when some others who had disdained my precaution were, figuratively speaking, tearing their hair and gnashing their teeth.

A Christmas Ward.

Nothing mars the pleasure of Christmas more than an irritable sister and fagged-out nurses, and though the latter cannot always be avoided, I think the friction caused by a feeling of inability to be "finished in time" can.

Moreover, it is all important to the general cheerfulness that every patient capable of doing so should help, and as it takes much less time to do a thing oneself than to teach unaccustomed fingers, it is wise to allow

In Hospital.

At Home.

oneself a wide margin as to time. It is not easy to acquire the art (especially where fingers are more accustomed to the use of pickaxe and shovel) of weaving nice round wreaths; there is a tendency in them to develop disastrous bald patches in unexpected places and melancholy lengths of feeble, attenuated greenery resembling wounded worms. Wreathing will not spoil in a fortnight if it is kept in a cool place. In October and November all flags can be reviewed and ironed, and if their tinsel-covered staves need renewing it can be done in a leisurely manner. All texts and mottoes will want renovating, and probably rebordering, and it is well to think out calmly all your intended little gifts.

### The Christmas Tree and Presents.

Our matron always provided a huge Christmas tree, and underneath its tinsel splendour, heavy with small gifts, sweetmeats, and ornaments, reposed large baskets of useful presents for every patient in the hospital. This involved a great deal of labour with which the sisters had nothing to do, beyond the agreeable task of choosing for our own patients and marking the articles with their names. Even this required much thought and tact, so as not to arouse jealousy or offence. It is more difficult in the male wards, but if nothing suitable can be found for a manly form, one can usually give pleasure by finding something for the wife or children, or among the bachelors for the sweethearts; this always causes great delight, and is a joke that never pales. Among other little pleasures, I had stockings for the boys under ten, then came entreaties to extend the favoured age to fifteen; at last the importunity became so great, that it had to be stretched to twenty, and even then those of twenty-five felt themselves hardly treated. My night nurse would pile them on after midnight, and the excitement in the morning beggars description, although my little offerings were by no means of a valuable nature. I was myself the happy recipient of many gifts.

It was as the laws of the Medes and Persians which altereth not, that all my patients should work with their fingers, either at knitting cross, stitch, or crochet, and I found it answered admirably; at first they scorned the idea, but soon became converted to my views, and patiently constructed many singular works of art. It was touching to see how patiently and laboriously a navvy with huge, knotted hands, would strive with a kettle-holder for his missus with the legend "Polly put the kettle on" legibly inscribed thereon; or a rather pessimistic pincushion, setting forth the dispiriting information that "all flesh is grass." Soldiers and sailors can nearly all work well, and their efforts were stupendous—waistcoats, braces, and slippers were favoured by the Army and Navy, and notably pincushions with ambitious legends executed in tinsel, to the effect that "Britons never will be Slaves," or "England expects every man to do his duty." A great many of these works of art became my property, and I had as many pairs of slippers as a popular evangelical curate; one dear old man, once a soldier, presented me with a magnificent pair, the splendour of which could not be surpassed, and which I treasure among my dearest possessions. The design was ambitious, and carried out according to a bold and original conception. The groundwork of military scarlet was adorned with forget-me-nots of a fine growth and striking colour, and on the toes, emblazoned in steel beads, divided equally between the two, he thus set forth his sentiments—

> "The rose is red,
> The violet's blue,
> My love is sweet,
> And she is you!"

There is a terseness and finality about it that many a timid lover might copy with advantage.

### The Dinner.

Christmas Day itself is a grand affair. Generally after morning service in the chapel the wards are thrown open to the inspection of visitors other than friends of the patients, who have the afternoon as their special time. Then comes dinner. We generally had turkeys sent by generous neighbours, and these lordly birds were carved in sight of the diners, which caused great interest. We asked one of the visiting staff to preside, physicians in the medical wards and surgeons in the surgical. These gentlemen were much taken aback to see the loaded plates that would disappear before the onslaught of their "cases." Naturally one could venture to be more reckless with the surgical patients in the matter of good cheer, though the staff looked dubiously at me when I assured them that an adequate dose of *Hanstus domesticus* would put all things right. I liked to reserve the oranges and cakes for tea another day, for after two plates of turkey and two of pudding the fine edge of appetite seemed a little blunted.

### The Entertainments.

Then come the evening entertainments. We never omitted the nigger show. What joy to see the awe-inspiring house-surgeon trying to consume cake between the pieces without too great an admixture of lampblack, with which his face would be so liberally bedaubed that a waggish member of the staff said, "Now I hope you have been conscientious and done yourself all over!" Year after year saw the ever-fresh delights of the *pas de quatre*, executed in nigger dress and very "disposedly," as Melville said of Queen Elizabeth's dancing—up and down the wards, and round and round the poor medicos had to go again and again, amid the most flattering applause. It boots not to tell of the overpowering fatigue of the nurses at the conclusion of Christmas.

One year, which had been extra hard in consequence of an influx of accidents, I was so thoroughly worn out that for the first and last time in my life I fainted. Willing hands placed me on an empty bed, and I soon came to myself; the next bed was occupied by a confirmed drunkard, a favourite, however, with us all. Peering at me over the edge of his bed, he thus delivered himself: "Well, sister, if I had not seen you about all day I should have thought you had been out on the spree; I am took that way myself sometimes."

Ten years is a large slice out of anyone's life, but I look back with great pleasure to it, and I would fain spend again a Christmas within the walls of what novelists call "the palace of pain." Thank God there is not so much pain there, or so much mental suffering, as there used to be; and I trust that residence amid the sick and suffering may act beneficially upon the characters of nurses as well as patients.

It is certain that no woman can lead such a life for ten years without being either a very much better woman or a very much worse. Let it be our daily prayer that they may be the former.

### Hospital Stamps.*

If it is true that those who usually succeed in life are those who think for themselves, where can parents begin the pleasant task of teaching their offspring to think with real profit better than by explaining to them the object of the sale of hospital stamps? How happy a lesson might be taught by many a devoted father and mother during the approaching Christmastide by means of these stamps, the second issue of which, consisting of four distinct impressions, with marked face value of one shilling, half a crown, five shillings, and ten shillings, has recently been placed before the public. Such questions as the boy or girl will bring forward lend themselves in a most exceptional degree to the framing of replies calculated to lead the young idea to think of the woes and needs of others.

"Will the stamps do for letters, mother?"

"No, my dear, they are different from the post office stamps, which are for the payment of taking letters and parcels from one place to another; the hospital stamps are sold to those who wish to benefit the great institutions in which are long wards containing beds for the poor people of the streets and poverty-stricken homes who are ill or who have met with accidents."

"How does the money get to the hospitals by selling the stamps?"

"It is in this way. A great number of the different stamps are printed, and from all who buy them the money is collected and put into one large fund, which in due course is divided between those hospitals that stand in greatest need of help."

"Then will the hospitals have to go without money unless the stamps are sold, mother?"

"Not exactly. Many people give regularly to the hospitals. They just send the money to the authorities direct, but very many persons do not give at all, and it is as an appeal to these that the stamps have been more particularly prepared. Now you my child, never gave anything till now, but by buying a five shilling and a half-crown stamp you have presented the hospitals with seven shillings and sixpence."

"And I have got two stamps as well."

"Yes, dear, you have something to show for your thought for others, and perhaps if you let your friends at school see your stamps they will also wish for some and in that way you will be helping forward a good cause."

"It's a good cause because we couldn't do without the hospitals, could we, mother?"

"Indeed we could not. Where would all the very poor sick, who cannot afford to pay for a doctor, go to if there were no hospitals? They are most necessary places, and none of us can do too much to relieve them of pressing debts, and put them on a sound footing."

### A Child's Petition.

I've a book of pretty pictures
  As ever you did see,
And the pictures they are fixtures,
  As roots of any tree.

It is not a useless scrap-book,
  Sought for the passing hour;
That's just pick'd up and then forsook
  Like some poor faded flow'r.

A book of really pretty Stamps
  It is I want to show;
And beg of you some timely grants,
  To make its volume grow.

There's dark and bright and red and blue
  Just like a rainbow clear;
And when you know the good they do,
  You cannot style them dear.

Each one you buy will gain a sum,
  For sick and lonely poor,
Wherewith to go and get undone
  Some accident severe.

Or ailment that for long has wrought,
  On sad and weary frame;
The dreary sting of sickness fought,
  And fought again in vain.

The Stamps are pretty memoirs, too,
  Of Queen Victoria's reign;
And of the Prince's purpose true,
  In gaining funds and fame. —C. C.

* Messrs. Simpkin, Marshall, and Co., 4, Stationers' Hall Court, London, E.C., have published some stamp albums and subscription books for these stamps, containing an autograph letter from H.R.H. the Princess of Wales and portraits of the Queen and the Prince of Wales, which can be ordered direct or through any bookseller, price 6d. bound in cloth, or 2s. 6d. bound in morocco. These books constitute an elegant Christmas present for anybody.

### Maggie's Misfortune.

Maggie always had an inquiring mind. Even as a tiny baby in arms it was said that she would never take the rubber teat of the feeding bottle without first glancing to see how much milk was in the annexed bottle. But that was a tale told by a funny uncle. Anyway, Maggie was really inquisitive. She was meddlesome and unsatisfied. She wanted to know too much, as so very many of our adult friends do. Her motto might well have been "Seeing's believing." There was none of the simple childlike trustfulness about Maggie when once an element of uncertainty had entered her small head. She was six years old, and a Board School girl, when one day, returning through the streets with several companions to her home, she thought she espied a damaged orange lying almost obscured behind a stack of empty cases that stood against the market hall wall. Very naughtily she stole round the cases, and tried to reach the coveted object with her fully outstretched hand. But her efforts were unavailing; the fruit was quite beyond touch. She was a self-willed little creature, and it didn't occur to her to go away and leave the prize without making a downright dash for it; she therefore put her shoulder to the cases and pushed and struggled with all her might. Suddenly something gave way, and the whole pile of boxes came tumbling down about her. One of the top cases fell upon her ankle, and so loud were her cries that a kindly market man, after seeing her bare foot, carried her off to a hospital.

Maggie's Misfortune.

Maggie's Good Fortune.

### Maggie's Good Fortune.

Once inside the big building, and washed and bandaged, and questioned, she was placed in a cosy cot that stood in a long ward containing more than a dozen other cots with little girls and boys in them all. Her mother came to see her, and Maggie couldn't for the life of her understand why she did not get sworn at and scolded as usual. It was difficult for her to realise that she had knowingly done wrong and also escaped a "ragging" from her parent. Maggie's home life was by no means what it ought to have been, yet the nurses "took to her wonderful," as Maggie's mother explained to a neighbour over a pot of beer one evening.

While Maggie was in hospital Christmas came and went. She did enjoy Christmas. It was the happiest time of her life; she said so. On Christmas Day morning the Sister gave each child in the ward a pretty present. Maggie's present was a beautiful doll with dress and bonnet and gloves and "everyfink else on." Her delight was unbounded, and might have been unrestrained had Nurse only answered her questions later on in the day. Maggie was still inquisitive, and after cuddling and petting her new toy, it struck her that she would like to know where it came from. Nurse, very properly, would not say whether it had come from *Truth* or whether it was the result of the efforts of the Ladies' Committee, who collect all manner of needlework and useful things. Maggie is a bonny bairn, and it is a great pity that her home training has not been strict enough to eradicate her little besetting sins.

### A Christmas Present: A True Hospital Story.

By a late Hospital Secretary.

It was getting late one December evening as I sat in my room in a well-known childien's hospital in New York. I was thinking over a busy day—of the things left undone till to-morrow, of——

But here my reflections were interrupted by a knock at the door and the announcement that a visitor, despite the lateness of the hour, was waiting to see me. "Would I see this gentleman—he was very sorry to call so late, and would not detain me long, &c." Glancing at the card I saw that my unexpected visitor was the Bishop of ——. The maid ushered him in, and as I rose to receive him I thought his manner nervous and hurried. We sat down by the glowing stove, for there was a sharp frost, and the snow outside anticipated the Christmas season. But the Bishop seemed somewhat at a loss to explain his visit. So we talked of other things—of those trivialities that invariably come uppermost in the mind when two persons are trying to break the conversational ice.

"The fact is," said the Bishop, with a sudden effort, and pulling himself together for the plunge. "I want a Christmas present for my wife, and I thought you might be able to help me."

To tell the truth I was completely mystified. The duties of a hospital secretary are multifarious and involved; but the day's work does not include the choice of episcopal and matrimonial Christmas offerings.

"I guess," said the American Bishop, noting my hesitancy, "I haven't made my meaning quite clear. I don't suppose this hospital to be a store of silver-mounted dressing-bags and conventional Christmas presents. It's a real live baby I want. The wife and I have never had a child, and I guess that's about the only thing she hasn't got. If you can help me to a pretty little girl baby, that will be about the most seasonable Christmas present I can take home."

The rest was plain sailing, for this was by no means the first time I had been asked to assist in the "adoption" of stranded infants. There is a beneficent law in New York that deserted or badly treated children become the legal property of the State. The parents lose all right from the fact of desertion, neglect, or cruel treatment. We had several such children in the institution, and possessed full power to "adopt out" any of these to selected foster parents able and willing to provide for them.

Directly the Bishop mentioned the word "baby" I thought of Ida. I never quite knew the full history of Ida—that was one point on which the dear old matron, who treasured enough true romances and stories in her head as the result of twenty years in our hospital, to make the fortune of a novelist, would never enlighten me. Ida, however, had been born in the hospital, and was "ours" to adopt out to the best advantage. Turning to the big books of record over which I presided, I found the date and place of her birth was clearly written —a blank space only marked the spot where particulars of parentage should have been. The previous secretary had died in the hospital; I was a newcomer, and beyond the date of Ida's birth could give the Bishop no information as to her family history. This much I knew, that her mother had been a gentlewoman. And Ida certainly looked as ladylike a baby as could have been found in any quarter of New York City.

I rang the bell, and sent a nurse for Ida. It was late to disturb the little girl, who was just a year old; but nameless babies rarely get the chance of an episcopal adoption, and I thought Ida in years to come would think such an opportunity quite worth the losing of an hour or so of beauty sleep. As we waited I fervently hoped that Ida would be at her best, and show off all the pretty little "points" which made her the prize baby among some 200 children sheltered in the institution. She had always been a clever little person, and her feminine wit served her to good purpose when she made her *début* before the waiting Bishop. Her charming little face was flushed with the excitement of being roused from her first sleep. The nurse who brought her in had sufficient wit to leave her tumbled hair in the pretty little ringlets of her pillow-tossed head. To "tidy" this would have robbed her of much of her charm. Altogether, she was a winsome little picture, and as she looked at the kindly Bishop out of wide blue eyes, I saw that she made a speedy conquest.

"I had not expected to find a child quite like this one," said the Bishop, afterwards, and I assured him that it was rare enough to have so aristocratic a foundling on our books; the most of our waifs and strays were of working-class parentage.

Ours was a curious institution. We had a maternity hospital, where all sorts and conditions of mothers sought refuge in their "sorrowful hour," as the Irish so quaintly put it. We had wards for acutely sick children and a nursery for healthy boys and girls whose parents were unable, through sickness, destitution, or dissipation, to care for them and bring them up properly. Deserted babies—foundlings from the doorstep and bye-ways of the city—were brought to us by the civic authorities, and for each such child a sum of ten dollars was paid monthly out of the public rates.

In his attitude towards Ida the Bishop was as much impressed by her outward and visible graces as any other man would have been. Probably he saw with episcopal vision the spiritual possibilities within—but I think her beauty prejudiced his judgment.

After satisfying us that he was truly and really the Bishop of ———, we arranged that Ida—"the wife's Christmas present"—should be sent to the household on a month's trial. Her charms were apparent; the Bishop's wife was anxious to discover whether these were of a "wearing quality," or only skin deep. A nurse was engaged for the trial visit, and Ida went off to her beautiful new home. She never returned. Her new parents were so charmed with her sweet nature and winning ways that by the end of a week they hurriedly took out papers of adoption, in terror lest somebody else in search of a daughter might attempt to annex their newly-found treasure.

Some months later I paid a visit to Ida, and found her reigning supreme as the "only daughter" of a charming country home. From an institution child—petted to some degree, even though there were so many to claim attention—she had blossomed into the daughter of a Bishop, clad in purple and fine linen. Her nursery was fit for a little princess, and she was the proud owner of a smart little pony cart devoted entirely to the use of "my daughter."

Ida is growing up now. She is receiving the education of a fashionable American "young lady." Her real mother has never been heard of, and, of course, even were she to apply at the institution she would not be supplied with any information as to her child's whereabouts. She lost claim for ever when she left the baby to the guardianship of the State. The good Bishop has made a will wherein he has provided for Ida just as though she were in reality his own child. He never ceases to congratulate himself on his successful choice of a "Christmas present." And Ida, were she to know what her possible future as an institution child might have resolved itself into, would never cease in her thankfulness to the instinct which led the Bishop on that particular evening to our hospital in search of a Christmas child. But Ida will never know. She recognises herself as an "adopted daughter," but neither she nor the Bishop's circle will ever be told of the "little business matter" that was talked over during the Christmas season in the sitting-room of a hospital secretary.

### SWEET CHARITY'S GUIDE TO CHRISTMAS GIVERS.

### GENERAL HOSPITALS.

**Charing Cross Hospital,** Agar Street, West Strand, W.C.—This institution is situated in the heart of the metropolis and in some of its most densely-populated and poor districts. Surrounded by crowded thoroughfares, it has to provide for an unusual number of accidents. The hospital has a convalescent home, containing 50 beds, situated on Limpsfield Common. £15,000 a year is required annually from voluntary sources to maintain the hospital and the home. The deficiency on the present year's working amounts to £3,000. Secretary, Mr. A. E. Reade.

**Great Northern Central Hospital,** Holloway Road, N.—Letters of recommendation are not required at this hospital. Over 1,600 in-patients are treated annually. The hospital is unendowed, and the reliable income is quite inadequate to meet the necessary expenditure—over £10,000 being required annually from voluntary sources. One ward containing 25 beds is still unopened for want of funds. Secretary, Mr. Lewis H. Glenton-Kerr.

**Guy's Hospital,** London Bridge, S.E.—At the present time Guy's Hospital requires the support of all its numerous friends in order that the great efforts which are being made to reopen its closed wards, and to bring the institution generally into the highest state of efficiency and usefulness, may be successfully brought to completion. One ward, with 32 beds, devoted to the reception of women suffering from diseases peculiar to their sex, was reopened in 1898. The net income of the hospital derived from its landed estates remains at £20,000 per annum less than it was before the great fall in rents of agricultural estates, and this depreciation must be regarded as permanent. Treasurer, Mr. H. Cosmo O. Bonsor, M.P. Superintendent, Dr. E. C. Perry.

**Hampstead Hospital,** Parliament Hill Road, N.W.—The number of in-patients in 1897 was 322, besides 190 minor accidents and casualties. Of the 305 in-patients 235 were free cases. There were also 1,789 attendances in the out-patient department. There is a heavy mortgage on the building which the council are most anxious to reduce. Hon. Secretary, Mr. R. A. Owthwaite.

**Italian Hospital,** Queen Square, Bloomsbury, W.C.—The old building having proved quite inadequate, and most unsuitable for the work which the hospital had to do, the committee have commenced building a new hospital on the old site and a site adjoining. Funds are urgently needed for the erection of the new hospital. Secretary, M. G. Ferrari.

**King's College Hospital,** Portugal Street, Lincoln's Inn, W.C.—2,604 in and 23,877 out-patients were treated in 1897, in addition to 633 poor married women attended during confinement in their own homes. Warden, Rev. N. Bromley.

**London Homœopathic Hospital,** Great Ormond Street, W.C.—The annual expenditure in the new building which was recently opened is £9,000. The income is only at the rate of £7,000 per annum. There is, therefore, an urgent need of an immediate and substantial increase in the annual subscription list. Treasurer, Lord Cawdor, 7, Princes Gardens, S.W. Secretary-Superintendent, Mr. G. A. Cross.

**London Hospital,** Whitechapel Road, E.—This is the largest voluntary hospital in this country. From this fact, and from the zealous manner in which the work is carried on, we make no doubt that the public will give largely to its funds, in testimony of their appreciation of the enormous value of what is being done there for the poor of East London. It is in serious want of funds, as the committee depend on voluntary contributions for £40,000 a year to enable them to maintain the 650 beds which are daily occupied by urgent cases. House Governor and Secretary, Mr. G. Q. Roberts, M.A.

**Metropolitan Hospital,** Kingsland Road, N.E.—The need for this hospital in the poor and densely populated districts in the midst of which it is situated is undoubted, but the charity is much crippled by lack of funds, and several beds have had to be closed, leaving only 70 now available for in-patients, although there is accommodation for 160. There is a debt at the present time of over £9,000 incurred during the past few years. Secretary, Mr. C. H. Byers.

**Middlesex Hospital,** Mortimer Street, W.—The in-patients for last year numbered 3,641, and the out-patients 47,252; the income from all sources, including legacies and a special donation to the Permanent Endowment Fund, was £42,145, and the expenditure £30,758; deficiency on the ordinary income and expenditure, £10,312. The cancer wards are a distinguishing feature of the hospital, and their extra nursing, costly treatment, and unlimited dietary add largely to the expenses of the hospital. Towards the extension of this department, by removing it from the main body of the hospital and building a new wing, £10,000 is still needed. Secretary-Superintendent, Mr. F. Clare Melhado.

**North-West London Hospital,** Kentish Town Road, N.W., was founded in 1878, and is the only institution of the kind in the north-west district. It has 53 beds, and last year there were 560 in-patients and 17,276 out-patients. Secretary, Mr. Alfred Craske.

**Poplar Hospital for Accidents,** Blackwall, E.—Situated amongst a teeming population of poor hardworking people in a district which may be called the "workshop" as well as the "port" of London, this hospital is doing an excellent work. The demands on this institution of late years have greatly increased, and consequently further subscriptions and donations are very necessary. Chairman, the Hon. Sydney Holland. Secretary, Lieut.-Colonel Feneran.

**Royal Free Hospital,** Gray's Inn Road, W.C.—Having no endowment, this hospital is entirely dependent for support on the subscriptions of its governors and the voluntary donations and bequests of its friends. It admits into its wards over 2,000 poor sick persons annually, besides administering advice and medicine to more than 25,000 out-patients who resort to it, not only from the crowded courts and alleys in the immediate neighbourhood, but from all parts of London and the surburban districts. The relief thus afforded is effected at a cost of about £12,000 per annum. Secretary, Mr. Conrad W. Thies.

**St. George's Hospital,** Hyde Park Corner, S.W.—This institution contains 351 beds, and during 1897 treated 4,689 in-patients, 15,518 out-patients, 12,096 casualties, and 404 maternity cases. Secretary and Superintendent, Mr. C. L. Todd.

**St. Mary's Hospital,** Paddington, W.—Very heavy work is thrown upon this institution owing to the large area of scattered poor which it has to assist. It is not so well supported as it should be, in spite of, or rather because of, its many wealthy neighbours. The board of management, therefore, urgently appeal for further support. The special wants of the hospital are: (1) New annual subscriptions; (2) donations for the endowment of beds and cots; (3) £65 in new annual subscriptions to the Maternity Fund to defray the cost of the maternity nurse. The hospital is free, and no urgent case is refused admission. Secretary, Mr. Thomas Ryan.

**St. Thomas's Hospital,** Westminster Bridge Road, S.E.—Since appealing to the public in 1895 the Governors have been able to reopen three of the five wards closed in 1871 for want of funds. It is, however, of great importance that the remaining two wards (at present used for paying patients) should be rendered available for the sick poor of South London, for whom so little hospital accommodation is provided. During the last 15 years the population of the district has increased at the rate of about 25,000 annually, and yet there are fewer beds available now than in 1871. A Bed Endowment Fund has recently been established, to which contributions of £1,000 are invited. Treasurer, Mr. T. Wainwright.

**Seamen's Hospital Society, "Dreadnought,"** Greenwich, S.E.—During 1897 representatives of over 50 different nationalities were admitted to the various establishments of the Society. The branch hospital at the Royal Victoria and Albert Docks is being enlarged, the accommodation there being inadequate to meet the requirements of that busy centre of shipping. In connection with this branch the London School of Tropical Medicine has been established. Secretary, Mr. P. Michelli.

**University College Hospital,** Gower Street, W.—The demands upon the resources of the hospital last year were very great, as the entire closing of a hospital in the immediate neighbourhood and the partial closing of two others have thrown a great strain upon the charity. The debt to tradesmen alone now exceeds £5,000. The committee point out in their Christmas appeal that the building of the new hospital by Sir J. Blundell Maple "will in no way whatever assist the Maintenance Fund." Secretary, Mr. Newton H. Nixon.

**Westminster Hospital,** Broad Sanctuary, S.W.—Out of an expenditure of £16,000, less than £3,000 is assured to this charity, so that about £13,000 has to be made up each year in subscriptions, donations, and legacies. The work of the hospital in 1897 included the treatment of 2,618 in-patients, 22,844 out-patients and casualties, and 257 lying-in women. The hospital does an immense service to the country by training a large number of excellent nurses. Secretary, Mr. Sidney M. Quennell.

### PROVINCIAL HOSPITALS.

**The Birmingham General Hospital.**—The new building of this hospital was opened by H.R.H. Princess Christian, on behalf of H.M. the Queen, on July 7th, 1897, and contains 340 beds. To meet the increased accommodation provided a larger income is necessary, and an earnest appeal is made for new and increased annual subscriptions. House Governor, Mr. Howard J. Collins.

**Bournemouth National Sanatorium for Consumption and Diseases of the Chest.**—Patients who are convalescent, those who require further medical treatment and change of air, and those suffering from an incipient form of the disease, are cases for the relief of which this institution was founded. There is accommodation for 31 men and 31 women, and about 300 patients from all parts of the kingdom are treated annually. The committee earnestly solicit liberal support, as, with the exception of a small dividend, the sanatorium is entirely dependent on voluntary contributions. Secretary, Mr. G. Lowe Riddett.

### SPECIAL HOSPITALS.

### CONSUMPTION.

**Brompton Hospital for Consumption and Diseases of the Chest,** S.W.—This hospital is well known as one where patients are well cared for, and every effort is made to brighten their clouded lives. The committee appeal for new subscribers to replace those removed by death, and for assistance to enable them to erect a much-needed country branch and a nurses' house on ground in rear of the hospital. Funds are also asked for towards the annual Christmas tree. Secretary, Mr. W. H. Theobald.

**City of London Hospital for Diseases of the Chest,** Victoria Park, E.—Situated in the East of London, where the diseases it treats are so common, about 1,000 in-patients and 15,000 out-patients are treated yearly. 60 beds are still closed owing to lack of funds, leaving only about 100 available for patients. Assistance is much required to meet current expenditure. Donations or annual subscriptions will be thankfully received. Secretary, Mr. Henry T. Dudley Ryder.

**North London Hospital for Consumption and Diseases of the Chest,** Mount Vernon, Hampstead, N.W., and Fitzroy Square, W.—This institution enjoys the advantage of a magnificent situation, where patients can obtain an abundance of pure fresh air, so necessary in the treatment of chest diseases. There is room in the hospital for 80 beds, but the present income does not admit of more than 60 being opened. Secretary, Mr. William J. Morton.

**Royal Hospital for Diseases of the Chest,** City Road, E.C.—The expenditure at this hospital exceeds £8,000, towards which there is an annual subscription list of some £2,250, and dividends amounting to £120. Two additional wards were opened last year, and funds are urgently needed. Donations will be gratefully acknowledged by the Secretary, Mr. John Harrold.

**Royal National Hospital for Consumption and Diseases of the Chest,** Ventnor, Isle of Wight.—Accommodation is at present provided for 133 patients (83 men and 50 women.) A new block for 21 additional men patients will be opened in 1899. As the expenses exceed the assured income by £4,000, the committee appeal for additional annual subscriptions and donations. Secretary, Mr. Ernest Morgan. London office, 34, Craven Street, Charing Cross, S.W.

### LYING-IN.

**City of London Lying-in Hospital,** City Road, E.C.—Funds are needed to carry on the general work of the hospital, and also to defray the cost of building new dormitories for the nurses and pupils. Secretary, Mr. R. A. Owthwaite.

**Queen Charlotte's Lying-in Hospital,** Marylebone Road, N.W.—This hospital received 1,101 patients in its wards last year, and in addition attended 1,124 patients at their own homes. To meet the great pressure on the accommodation of the hospital an extension is being carried out, and a new nurses' home is in course of erection. For these works upwards of £5,000 is still needed. Donations to the building fund, as well as for general maintenance, are earnestly solicited. Secretary, Mr. Arthur Watts.

### EPILEPSY AND PARALYSIS.

**National Hospital for the Paralysed and Epileptic** (Albany Memorial), Queen Square, W.C.—The total number of beds provided at this hospital and its Finchley branch is 200, but the accommodation is still painfully insufficient to meet the requirements, as the large majority of patients are unsuited to general hospitals. Many urgent cases are always waiting for beds. Besides the hospital for treatment, there is a pension fund for the incurables. The annual expenditure is £16,000, of which £10,000 must be raised in benefactions. Director, Mr. B. Burford Rawlings.

### CHILDREN.

**Alexandra Hospital for Children with Hip Disease,** Queen Square, Bloomsbury, W.C.—The necessity for this hospital for hip disease may best be shown by the fact that nearly three-fourths of its patients come from other London hospitals, the reason being that no general hospital can retain a patient for two or three years as is not infrequently done in this hospital. The hospital is absolutely unendowed, and depends solely upon voluntary contributions. £1,000 is urgently needed for maintenance, and £5,000 to complete the new hospital now in course of erection. Secretary, Mr. Stanley Smith.

**East London Hospital for Children and Dispensary for Women,** Shadwell, E.—It will readily be understood that the struggle for existence in such a neighbourhood as Shadwell is very great, and for this reason the committee of the East London Hospital for Children appeal to the residents in wealthier districts for funds to enable them to help those who are unable to help themselves. The seaside branch (28 beds) for convalescent patients which the committee have just opened at Bognor, while materially increasing the usefulness of the charity, will at the same time increase its need of support. Secretary, Mr. Thomas Hayes.

**North-Eastern Hospital for Children,** Hackney Road, Shoreditch.—This hospital, placed in the midst of one of the most crowded districts of East London, is too small for the work it is called upon to do, and is sadly in need of enlargement. The committee are, therefore, trying to obtain £25,000 for this purpose, and have succeeded in raising £10,200. Help is also needed for maintenance. Secretary, Mr. T. Glenton-Kerr. City office, 27, Clement's Lane, E.C.

**Paddington Green Children's Hospital.**—This hospital was rebuilt and enlarged in 1894. In order to complete it the committee were compelled to borrow £2,300 from the bankers, and they now earnestly appeal for contributions to enable them to repay this loan before the end of the current year. Secretary, Mr. W. H. Pearce.

**The Hospital for Sick Children,** Great Ormond Street, W.C.—This hospital, which is the largest children's hospital in the kingdom, still requires £20,000 of the £30,000 which has to be expended in order to enable this institution to purchase the adjoining Hospital and Convent of St. John and St. Elizabeth. They also require £500 a year in new annual subscriptions to meet the continued loss of income under this head. Secretary, Mr. Adrian Hope.

**Victoria Hospital for Children,** Queen's Road, Chelsea, S.W. (and Victoria Convalescent Home, Broadstairs). —The committee are appealing for special donations to the fund for the erection of a new building at a cost of £25,000. The hospital further seeks subscriptions to help it to carry on its work. Secretary, Mr. A. Cameron Skinner.

### WOMEN.

**Chelsea Hospital for Women,** Fulham Road, S.W. —This hospital (52 beds) is entirely without endowment or reserve funds, and greatly needs legacies and new annual subscriptions. There is a Convalescent Home (22 beds) at St. Leonards-on-Sea, but it is not restricted to those who have been patients in the hospital. Secretary, Mr. Herbert H. Jennings.

**Grosvenor Hospital for Women and Children,** Vincent Square, Westminster, S.W.—This hospital, hitherto inadequate and ill-contrived, has been recently reconstructed with modern improvements. Increased support is needed, two wards being still closed for want of funds. Secretary, Mr. W. Aston-Lewis.

**New Hospital for Women,** 144, Euston Road, N.W. —Only a want of funds prevents the committee from providing the beds necessary to treat the numerous cases waiting for admission to this hospital. Each bed costs about £75 a year, and an appeal is made to generous friends to come to the aid of the committee by endowing beds with an amount that would produce this annual sum. Secretary, Miss M. M. Bagster.

**Royal Hospital for Children and Women,** Waterloo Bridge Road, S.E.—375 in-patients and 6,525 outpatients were treated during 1897. Secretary, Mr. Thos. S. Conisbee.

**Samaritan Free Hospital for Women and Children,** Marylebone Road, N.W.—Some £7,000 per annum is required to maintain this hospital, of which only £1,700 can be relied upon in annual subscriptions. The committee would, therefore, like to see a great addition to the list of annual and life subscriptions. Secretary, Mr. G. Scudamore.

**The Hospital for Women,** Soho Square, W.—There are 61 beds in constant use, and, as the hospital possesses no endowment, funds for their maintenance are much needed. The committee very earnestly appeal for additional annual subscriptions. Secretary, Mr. David Cannon.

### MISCELLANEOUS—SPECIAL.

**Dental Hospital of London,** Leicester Square, W.C. —The present hospital, which has been enlarged to its utmost extent, is quite inadequate, both in accommodation and sanitation, for the large number of patients. A new hospital is, therefore, being built, and funds, for which an appeal has for some time been before the public, are still urgently needed. Secretary, Mr. J. Francis Pink.

**London Lock Hospital and Home,** Harrow Road, W.—Funds are much wanted to meet heavy expenditure on the upkeep of the buildings, one item of this expenditure being rendered necessary by the subsidence of one end of the hospital, necessitating the putting in of new foundations at once. Secretary, Mr. A. W. Cruikshank.

**London Fever Hospital,** Liverpool Road, Islington, N.—The institution is dependent upon voluntary support, and donations and subscriptions will be gratefully received, especially as the alterations and additions to the hospital, now being carried out, and the building of a convalescent home, are taxing to the utmost the resources of the institution. Secretary, Major W. Christie.

**National Orthopædic Hospital,** 324, Great Portland Street, W.—The hospital's most pressing need is to raise £400 before the end of the year to prevent a deficit. There is still a debt of £1,300 on the building, and the managers wish to increase its list of annual subscribers to three times its present amount. Secretary, Mr. H. J. Tresidder.

**Royal Eye Hospital,** St. George's Circus, Southwark, S.E.—The work of this hospital increases year by year, and in 1897 15,848 new cases were treated. Secretary, Mrs. T. E. Cope.

**Royal London Ophthalmic Hospital,** Moorfields, E.C.—An urgent appeal is made by the board of management for funds in support of this institution. The patients numbered in 1897: In-patients, 1,968; out-patients, 25,051. All patients are admitted free, and without letters of recommendation. Secretary, Mr. Robert J. Bland.

**Royal Orthopædic Hospital,** 297, Oxford Street, W. —The governors in their last report state that unless increased help is soon forthcoming it will be impossible to keep the whole of the 50 beds open. As there are at present about 50 patients waiting for admission such a step would be most deplorable. Secretary, Mr. Tate S. Mansford.

**St. Luke's Hospital,** Old Street, E.C.—Very extensive repairs are still being carried out to the external walls. These repairs have been absolutely necessary, seeing that the building was erected over 110 years ago. The treatment of mental diseases is a special work, and must appeal strongly to the average man and woman. St. Luke's Hospital has been treating such patients for over 150 years; is now entirely devoted to our great "middle-class population," whose means are limited, and for whom there is no other relief but by becoming chargeable to the poor-rates. Over £6,000 has had to be spent from the funds on building charges during the last three years. Thus help is very urgently needed to maintain the charity. Secretary, Mr. W. H. Baird.

### GENERAL CHARITIES.

**Association for the Oral Instruction of the Deaf and Dumb.**—The first association to publicly introduce into the United Kingdom the German or pure oral system for teaching deaf and so-called dumb children to speak *viva voce*, and to understand the spoken words of others by lip-reading. The expenses, which are very heavy, are met by voluntary contributions and fees, which fall short of what is needed by about £700 per annum. Offices, 11, Fitzroy Square, W.

**Bethnal Green Free Library, E.**—The poverty in the surrounding neighbourhood compels the committee to make an urgent appeal for funds to meet outstanding liabilities. Contributions will be thankfully received by the Librarian, at the Bethnal Green Free Library, E., or by the Treasurer, Mr. F. A. Bevan, 54, Lombard Street, E.C.

**British Home for Incurables,** Crown Lane, Streatham Common, S.W. Instituted in 1861.—This home is intended for those who, having once been in a position of comfort, are now in necessitous circumstances, and are either bedridden or greatly dependent on others in the various offices of life. For these the institution provides medical attendance, nursing, and the comforts of a home for life. Annuities of £20 are conferred upon such as are incurable, but not wholly destitute, in order that they may continue to reside with friends or relatives, who may be able to render them some further assistance. Secretary, Mr. R. G. Salmond, 72, Cheapside, E.C.

**City of London Truss Society,** 35, Finsbury Square, E.C.—At the present time about 10,000 of both sexes and all ages are treated annually. The great growth in the relief afforded by the society in recent years is shown by the fact that of the 538,000 patients treated during the ninety-one years of its existence nearly one-half have been relieved within the last twenty-six years. Secretary, Mr. John Whittington.

**Irish Distressed Ladies' Fund,** 17, North Audley Street, W.—Relief is given independently of any question of politics or religion; employment is found, when possible, for those able to work; pecuniary help is given to the aged and infirm; and the education of children is paid for.

**London Orphan Asylum,** Watford.—The managers of this institution make an especial appeal at this season, and plead that an institution which in the past 85 years has maintained and educated 6,080 respectable fatherless children, and which has at the present moment 500 boys and girls under its care, should appeal most eloquently to the sympathy of the benevolent. The maintenance of the work in its present efficiency involves an expenditure of over £15,000 per annum. The managers have had to borrow £2,000 to meet the current year's expenses, and they, therefore, appeal for help in clearing off this debt and in meeting the coming year's liability without recourse to borrowing or the painful expedient of reducing the number of children benefited. Secretary, Mr. Henry C. Armiger, 21, Great St. Helen's, E.C.

**London Schools Dinner Association.**—Established in 1889, to provide cheap or free meals for necessitous children attending the public elementary schools of London, this association is doing an excellent work. The annual income now averages about £1,500, but it is reckoned that double that sum is necessary to meet the needs of the children. Secretary, Mr. T. A. Spalding, 37, Norfolk Street, Strand, W.C.

**London Society for Teaching the Blind,** 10, Upper Avenue Road, Hampstead Road, N.W.—This society educates children, blind and partially blind, in general and technical instruction, viz., music, piano tuning, basketmaking, needlework, &c. Both sexes and all denominations received. The cost of carrying out this work is £2,800 a year, and the income of this society falls short of this amount by £600. Secretary, Captain G. G. Webber, R N.

**Mary Wardell Convalescent Home for Scarlet Fever,** Stanmore, Middlesex.—This being the only home for convalescents from scarlet fever, the demand on its 40 beds is great. Funds are greatly needed to meet the yearly expenses, and the debt unavoidably incurred in renovating the home has not yet been liquidated. Subscriptions to Miss Mary Wardell.

**Metropolitan Convalescent Institution,** 32, Sackville Street, Piccadilly, W.—This institution has three convalescent homes, one (for men and women) at Walton, another (for children) at Broadstairs, and the third (for men and women) at Bexhill-on-Sea. Additional subscriptions for the maintenance of the homes, and donations for the completion of the children's branch (for which about £6,000 is required), are appealed for. Secretary, Alexander Hayes.

**Orphan Working School,** Haverstock Hill, N.W., and Hornsey Rise, N. Offices, 73, Cheapside, E.C.—A national and undenominational institution which maintains 500 children, varying in age from infancy to fourteen or (in special cases) fifteen years. It is greatly in want of funds at the present time, urgently needed to meet immediate and pressing liabilities. Secretary, Mr. Algernon C. P. Coote, M.A.

**Royal Albert Orphan Asylum,** Bagshot.—This institution affords a home and industrial training for about 200 fatherless children. No canvassing or sale of votes is permitted. Help is urgently needed, as the debts are heavy, and a curtailment of the work will be necessary unless funds are forthcoming. Secretary, Mr. H. W. Tatum. Office, 62, King William Street, E.C.

**Royal Association in Aid of the Deaf and Dumb,** Oxford Street, W.—The objects of this society are to visit deaf and dumb persons at their own homes, and to assist them in sickness and distress. During the past year 3,422 visits were paid to the deaf and dumb, 1,033 visits were made to employers and others on their behalf, 206 were relieved, and 63 provided with work and apprenticed. An increased reliable income is needed to maintain and extend the present work. Secretary, Mr. Thomas Cole.

**Royal Blind Pension Society,** 237, Southwark Bridge Road, S.E.—This society provides pensions, by monthly instalments, for 1,009 blind people. Further contributions, particularly annual subscriptions, are required to enable it to give aid to 200 candidates who are approved as eligible for charitable assistance. Secretary, Mr. W. E. Terry.

**Royal National Lifeboat Institution,** 14, John Street, Adelphi, W.C.—295 Lifeboats are maintained by the society, and the committee earnestly appeal for funds to maintain them and their crews in a state of efficiency. The ordinary annual subscriptions, donations, and dividends are quite inadequate for the purpose. Secretary, Mr. Charles Dibdin.

**Royal Sea Bathing Hospital,** founded at Margate 1791.—The hospital has during the last few months been reconstructed and fitted with modern improvements at a cost of nearly £10,000. The full number of beds (150) will be available early in the year. At present only 60 are occupied. Secretary, Mr. A. Peirce, 30, Charing Cross, S.W.

**Shipwrecked Fishermen and Mariners' Royal Benevolent Society,** 26, Suffolk Street, Pall Mall East, S.W.—The work done by this society includes the granting of relief to shipwrecked persons, to widows and orphans of members and others, to destitute seamen, &c.; grants towards meeting loss of clothes or boats, assistance in old age or in case of infirmity. Over £19,000 was expended in relief in 1897. Secretary, Mr. Gerald E. Maude.

**Surgical Aid Society,** Salisbury Square, E.C.—The net expenditure for the year amounted to £12,780, of which 84 per cent. was for actual relief. During the past year the large total of 23,621 appliances were given away. There is ample scope for very considerable extension of these benefits, and, therefore, the committee earnestly appeal for contributions. Secretary, Mr. Richard C. Tresidder.

### Our Illustrations.

### IN HOSPITAL.

There are those who dread the thought of entering a hospital. To go even as a visitor is against their liking, for they possess a sort of dread of the place wherein the sick and helpless lie day after day in suffering. And there are others to whom a hospital is the one place of all others to which their heart's desire would take them. At many of the large London hospitals it could be ascertained that some of the patients have left the building with considerable reluctance. The comfort, and kindness, and system of the place have impressed them, and, having once overcome the feeling of strangeness, they have come to appreciate the good results of order and method. It is not at all an unknown thing for a Sister to be asked by an ex-patient for permission to visit the ward for the sake of auld lang syne. Each metropolitan hospital, at any rate, has its little army of eager outside workers; one brings a dozen small bunches of flowers every week, another brings a parcel of suitable literature, another contributes regularly to the Samaritan alms-box, and another, who lives by the sea, arranges to take a convalescent case now and then for a short period. A far-reaching power for good, above and beyond all medical and surgical remedies, lies within reach of a hospital as a whole, and we should be thankful that this power is seldom, if ever, allowed to slip by unused. Nowadays, when freedom of thought and creed are not looked too harshly upon, it is not difficult for the man or woman of the most individual notions to find a friend within the hospital walls, and to the little children who, alas! too frequently come from darkened homes and surroundings, the big, kindly hospital is indeed a great harbour of refuge.

### AT HOME.

The word "home" has been referred to by writers of various types as one of the sweetest sounding words in the English language. The word "mother" is another that is suggested as also conveying what is tender and true and much else. Happily, to most of us, home is the one word above all others that appeals to us when, either for the purpose of special business or pleasure, it has been necessary to leave behind for a period the accustomed place and faces. What youth, at the outset of a new career, or sailor, or soldier, or governess, or nurse, who has gone out into the world, does not hear or think of the one short, simple word "home," without at once picturing a circle of happy visions and memories? But not to all is the word "home" a diamond in language. Is there a brutal father, or a drunken mother, or a profligate son, or a wayward daughter? Then how often is the whole influence of good perverted, and sometimes for ever lost. To how many does the word imply nothing but a bare room or two, with an entire blank so far as love and comradeship are concerned. Where there is a blow instead of a caress, a blasphemy instead of a smile; where there is an empty cupboard and a lack of bare house furniture; where there is no greeting and no welcome, no heart and no hope. Our illustration suggests to the sympathetic eye all manner of possibilities, and the chief is neglect; home and children bereaved of the mother's and the father's care, and little hands that would be best at play are held up to hide the tear.

## Figures and Tables

**Figure f1:**
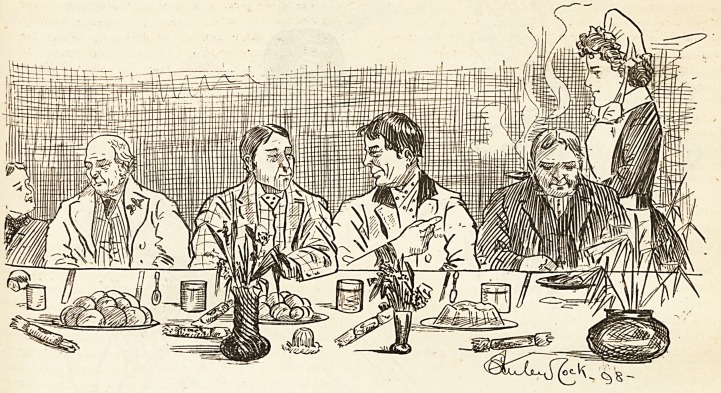


**Figure f2:**
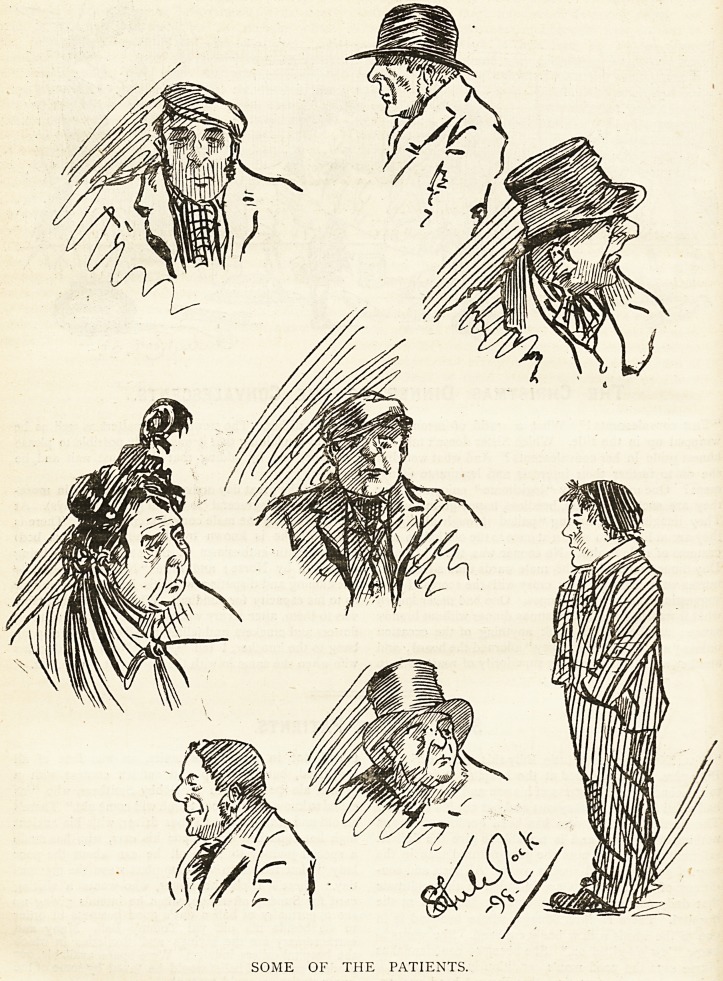


**Figure f3:**
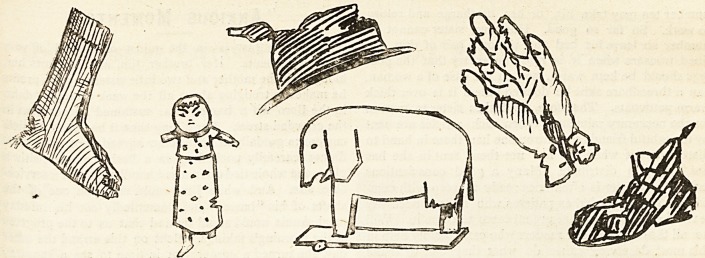


**Figure f4:**
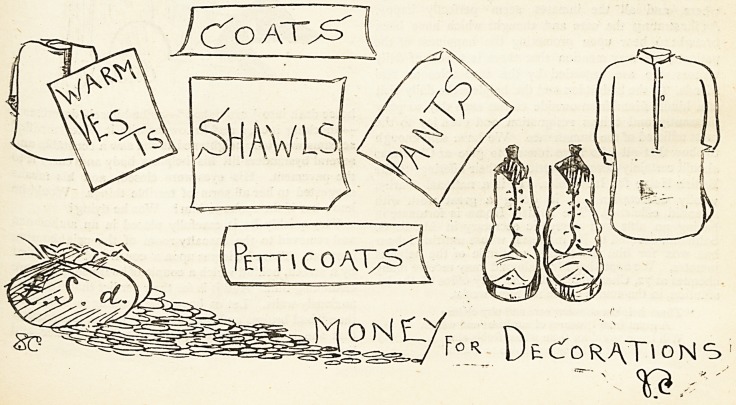


**Figure f5:**
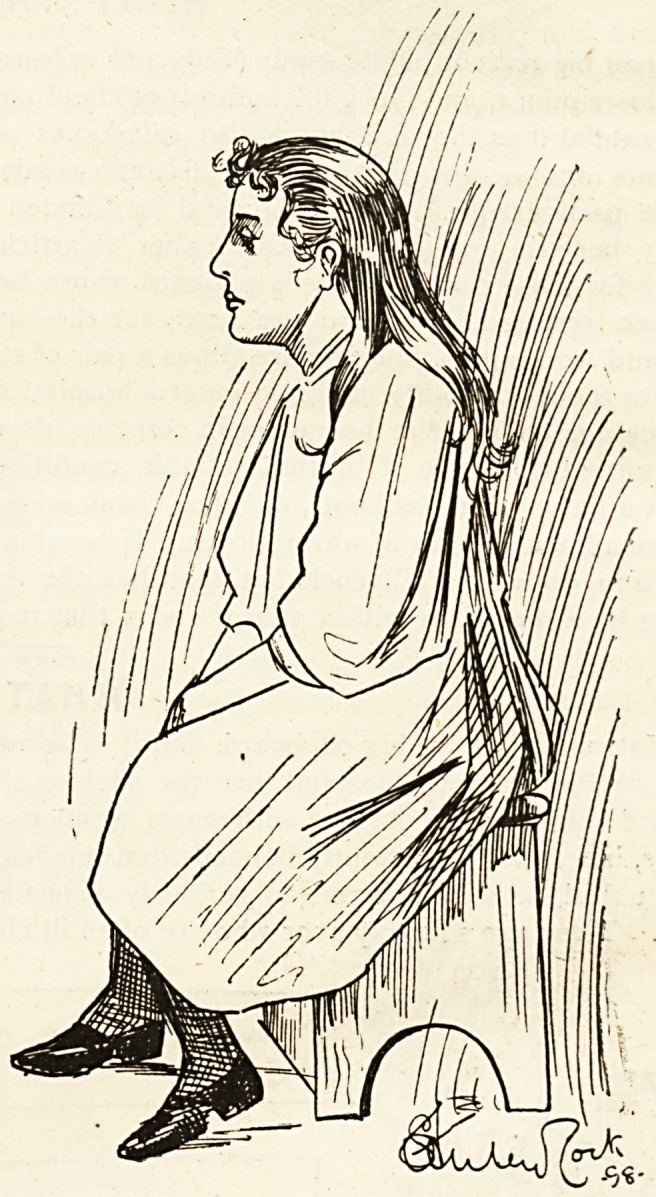


**Figure f6:**
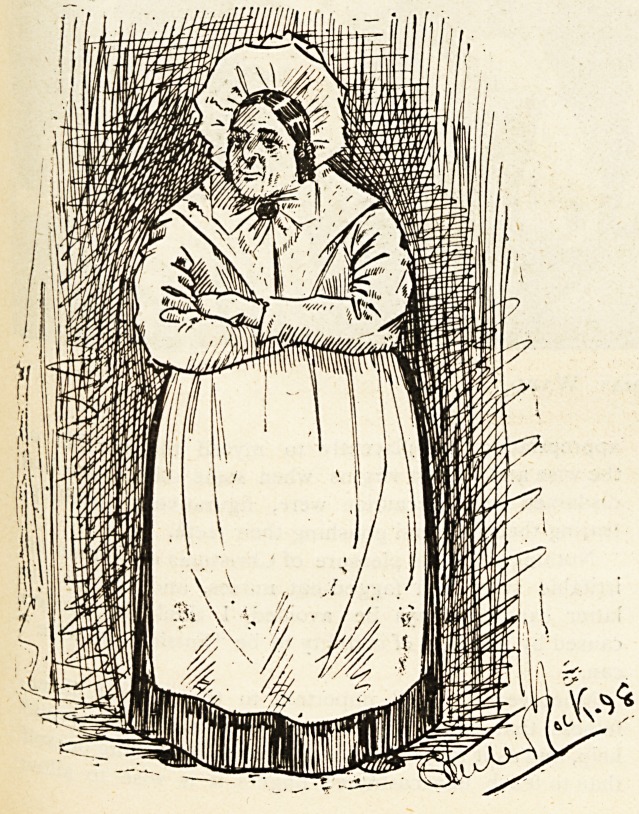


**Figure f7:**
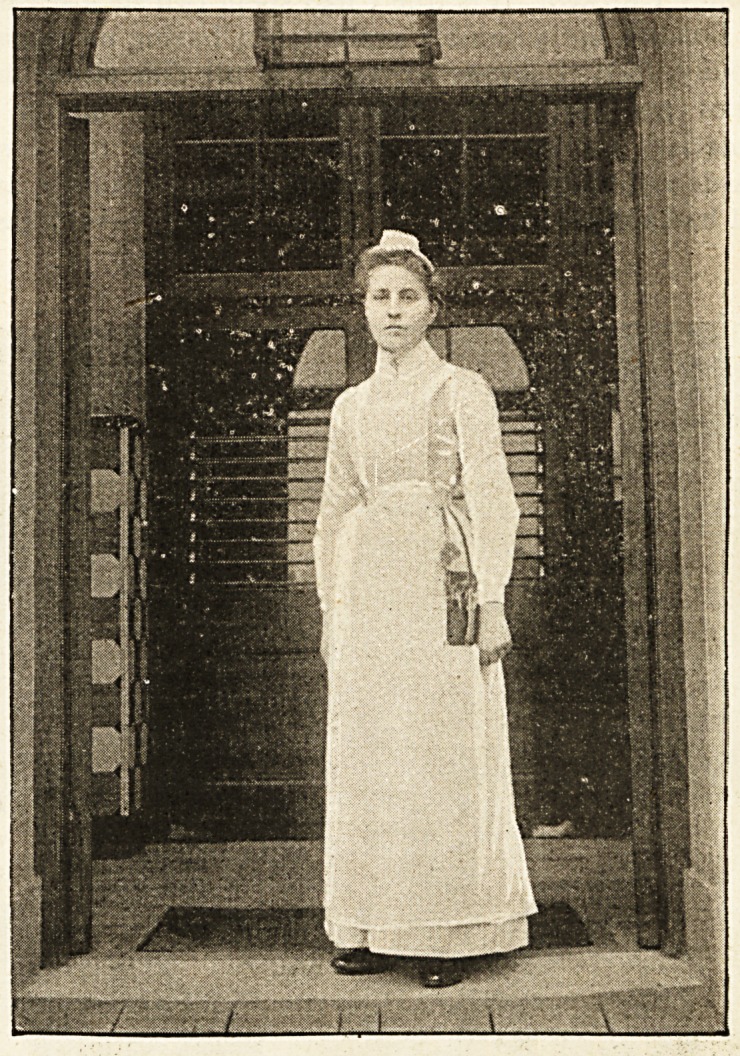


**Figure f8:**
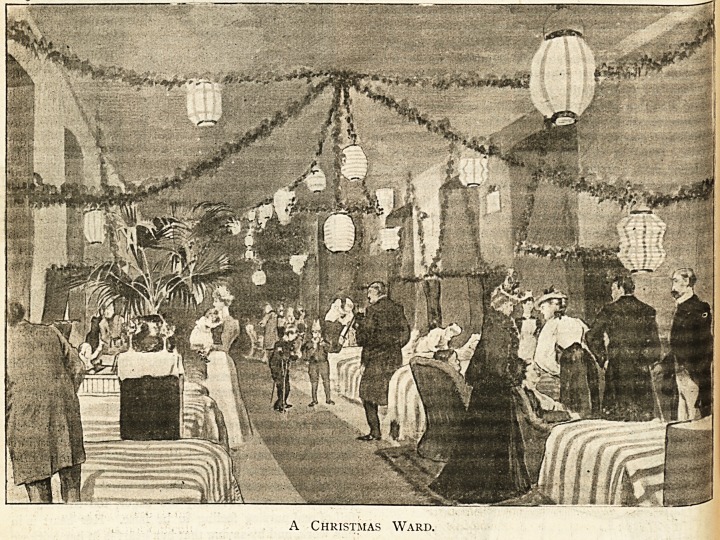


**Figure f9:**
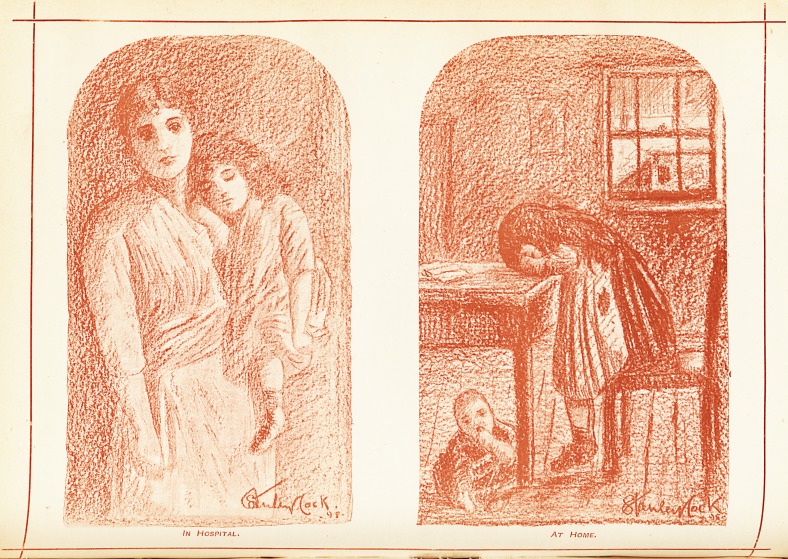


**Figure f10:**
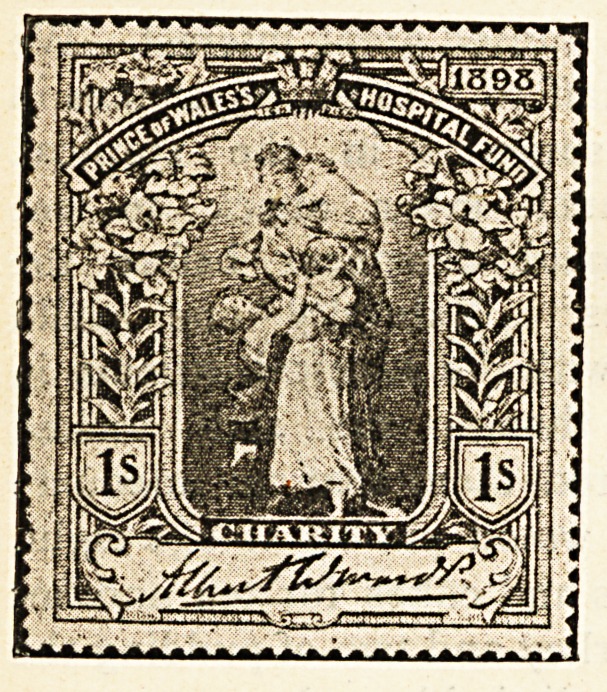


**Figure f11:**
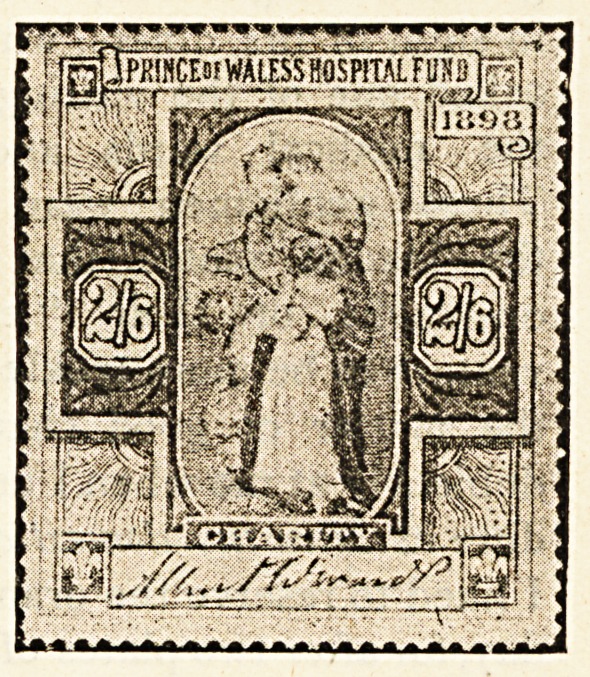


**Figure f12:**
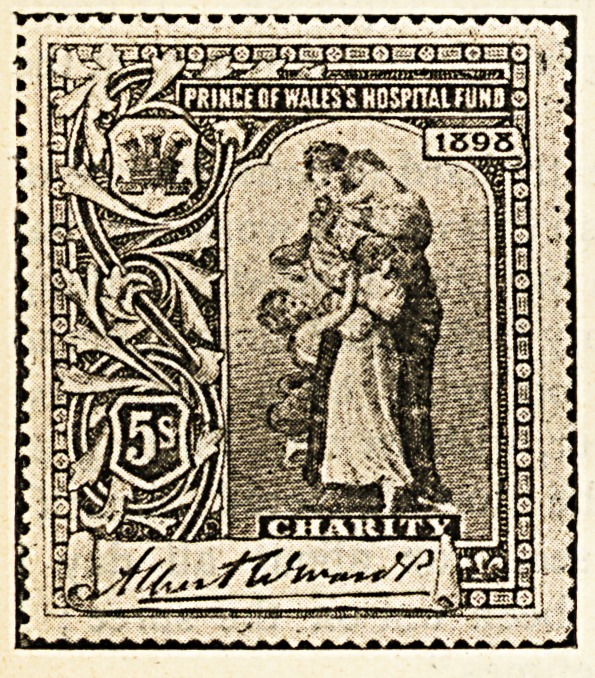


**Figure f13:**
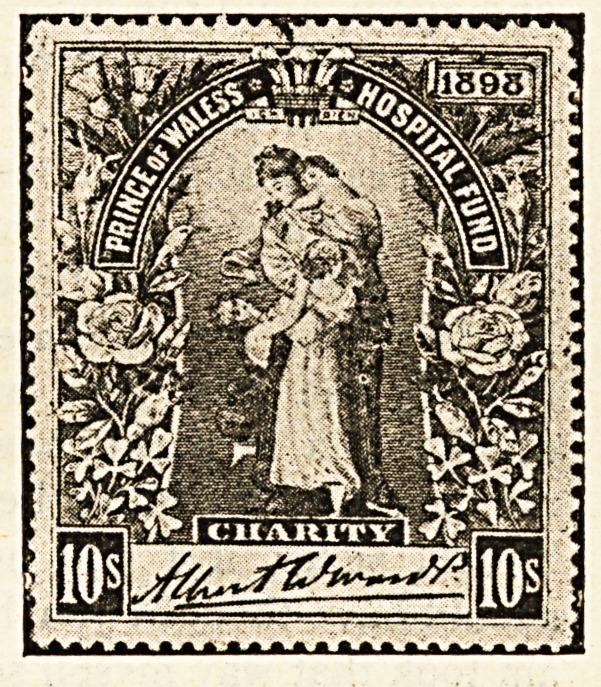


**Figure f14:**
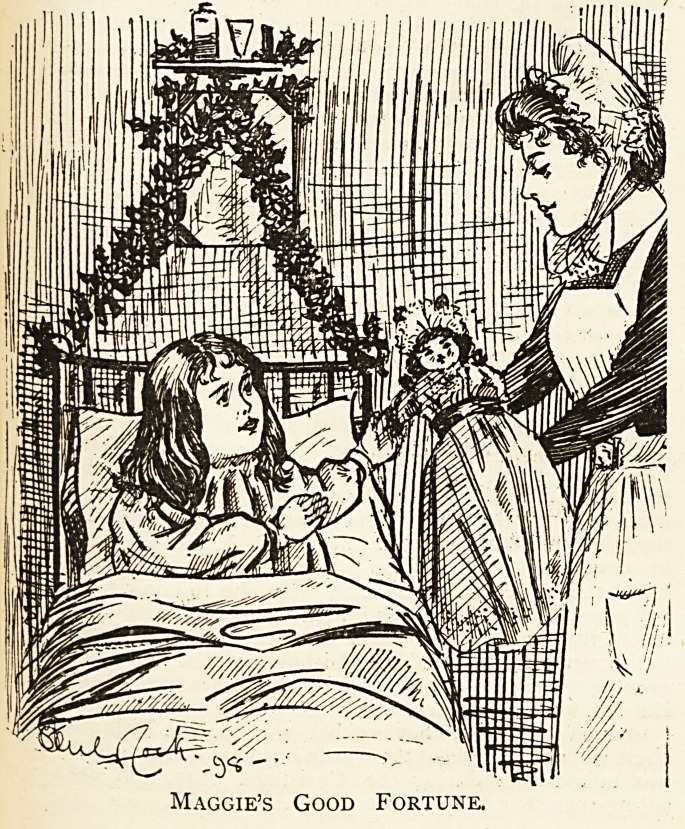


**Figure f15:**